# Extinction vulnerability of coral reef fishes

**DOI:** 10.1111/j.1461-0248.2011.01592.x

**Published:** 2011-04

**Authors:** Nicholas A J Graham, Pascale Chabanet, Richard D Evans, Simon Jennings, Yves Letourneur, M Aaron MacNeil, Tim R McClanahan, Marcus C Öhman, Nicholas V C Polunin, Shaun K Wilson

**Affiliations:** 1 ARC Centre of Excellence for Coral Reef Studies, James Cook UniversityTownsville, Qld 4811, Australia; 2 Institut de Recherche pour le Développement (IRD)BP 50172. 97492 Ste Clotilde Cedex, Reunion Island, France; 3 Department of Environment and Conservation17 Dick Perry Ave., Kensington, Perth, WA 6151, Australia; 4 Oceans Institute, School of Plant Biology, University of Western AustraliaCrawley, WA 6009, Australia; 5 Centre for Environment, Fisheries and Aquaculture Science, Lowestoft Laboratory, Lowestoft NR33 0HT, and School of Environmental Sciences, University of East AngliaNorwich NR4 7TJ, UK; 6 Laboratoire LIVE, Université de la Nouvelle-CalédonieBP R4, 98851 Nouméa Cedex, New Caledonia; 7 Australian Institute of Marine SciencePMB 3, Townsville MC, Townsville, Qld 4810, Australia; 8 Marine Programs, Wildlife Conservation SocietyBronx, NY 10460, USA; 9 Department of Zoology, Stockholm UniversityS-106 91 Stockholm, Sweden; 10 School of Marine Science and Technology, Newcastle UniversityNewcastle-upon-Tyne NE1 7RU, UK

**Keywords:** additive effects, climate change, coral reef ecology, ecosystem function, fisheries, multiple stressors, resilience, synergy

## Abstract

With rapidly increasing rates of contemporary extinction, predicting extinction vulnerability and identifying how multiple stressors drive non-random species loss have become key challenges in ecology. These assessments are crucial for avoiding the loss of key functional groups that sustain ecosystem processes and services. We developed a novel predictive framework of species extinction vulnerability and applied it to coral reef fishes. Although relatively few coral reef fishes are at risk of global extinction from climate disturbances, a negative convex relationship between fish species locally vulnerable to climate change vs. fisheries exploitation indicates that the entire community is vulnerable on the many reefs where both stressors co-occur. Fishes involved in maintaining key ecosystem functions are more at risk from fishing than climate disturbances. This finding is encouraging as local and regional commitment to fisheries management action can maintain reef ecosystem functions pending progress towards the more complex global problem of stabilizing the climate.

## Introduction

Contemporary extinction rates are rising, driven by direct anthropogenic pressures and forcing of the climate (Soulé 1991; Purvis *et al.* 2000; Novacek & Cleland 2001). The loss of species, including those that were previously abundant or maintained a critical function, can change the structure and stability of ecosystems (Hooper *et al.* 2005; Gaston & Fuller 2007). Proactive management of endangered species is reliant on our capacity to identify how key stressors interact to effect species abundances (Brook *et al.* 2008) and to develop predictive assessments of species extinction risk (Purvis *et al.* 2000; Dulvy *et al.* 2003). However, although extinctions are typically non-random (Purvis *et al.* 2000), predictive capacity is currently weak, particularly for marine organisms (Dulvy *et al.* 2003).

Early attempts to predict extinction vulnerability incorporated information on species geographical range size, occupancy (presence in habitats) and local numerical rarity (Rabinowitz 1981). However, as our understanding of species and their ecological versatility has grown, data on various forms of ecological specialization (Julliard *et al.* 2003), body size (Owens & Bennett 2000) and other life-history traits (Cheung *et al.* 2005) have been included in assessments of extinction risk. Specialization, body size and life-history traits help determine the likelihood that a species will undergo local losses and population declines following disturbances, whereas information on range size, occupancy and rarity indicates whether declines may lead to global extinction. Clearly these two types of information address different issues and spatial scales and, rather than be combined in a single composite indicator, may be used in parallel to provide a comprehensive assessment of species risk of local and global extinction.

Coral reefs are among the first ecosystems to show marked ecological responses to climate warming and variability (Hoegh-Guldberg *et al.* 2007). Coral reefs are also heavily impacted by direct human use, principally from heavy fishing that in many countries, targets a large proportion of the total fish assemblage (Polunin & Roberts 1996). These ongoing threats present an urgent need to assess the extinction vulnerability of reef-associated organisms to multiple stressors. Coral reef fishes are an essential ecological group because they form the principal link between reefs and associated human societies (Cinner *et al.* 2009) and because they play key roles in sustaining the ecological processes and functioning of reef ecosystems (Bellwood *et al.* 2004). Previous assessments of extinction vulnerability in reef fishes have focused on fisheries exploitation (Cheung *et al.* 2005). However, habitat disturbance, largely driven by climate warming and variability, also increases extinction risk for many species (Pratchett *et al.* 2008) and, in some instances, has led to greater decreases in fish abundance than fisheries exploitation (Wilson *et al.* 2008). Understanding differential vulnerability to fishing and climate change is necessary for determining how reef communities will respond where these two major stressors occur concurrently (Vinebrooke *et al.* 2004).

In this study, we developed a novel framework that partitions the vulnerability of populations to decline from the likelihood of declines causing global extinction. The framework can easily be applied to various organisms and disturbances. We use the approach to determine local and global vulnerability of 134 species of coral reef fishes, belonging to four major families and eight functional groups, to habitat degradation driven by climate change. We test the population decline predictions of our framework using a reef fish dataset that spans the largest climate-induced coral bleaching event recorded to date, the 1998 El Niño event. We further assess the reef fish community response to the combined effects of the two most pervasive stressors on coral reefs; climate change and fisheries.

## Material and methods

**Figure 1:**
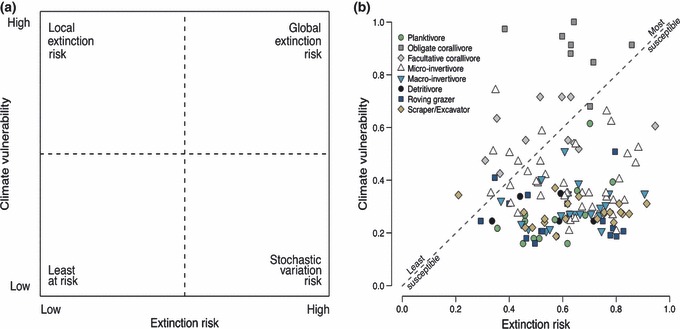
Predictive framework for assessing extinction risk. (a) The vulnerability of coral reef fish species populations to climate change disturbances (coral bleaching and mortality) against the risk that population decline is catastrophic and leads to species extinction. The vertical axis is an expert-weighted composite index including information on species specialization on coral and reef habitat for (1) diet, (2) habitat use and (3) settlement, and also including information on (4) species body size. The horizontal axis is an expert-weighted composite index of extinction risk and includes information on (1) geographical range size, (2) depth range, (3) occupancy and (4) numerical rarity. (b) Species data applied to the framework for 134 species of coral reef fishes in eight functional groups.

We developed a predictive bivariate approach to assess species vulnerability to extinction through climate change associated coral reef disturbance (Fig. 1a). With this framework, a species’ vulnerability to population declines following a climatic disturbance event (termed ‘climate vulnerability’) is plotted against the intrinsic extinction risk of that species (termed ‘extinction risk’). Based on scientific theory and published empirical assessments, four variables were included in the climate vulnerability index that are known to relate to population declines following benthic disturbances; diet specialization, habitat specialization, recruitment specialization for live coral and body size. Dietary specialization increases the likelihood of coral reef fish population decline following coral mortality, with obligate corallivores declining proportionately more in relation to their degree of specialization (Pratchett *et al.* 2006). Similarly, habitat specialization influences the extent of population decline following coral loss (Munday 2004). Many fish are also heavily dependent on coral during settlement and early life history (Öhman *et al.* 1998) and this fact has been shown to be critical to fish population declines following reef degradation (Jones *et al.* 2004). Finally, small-bodied fish species are often closely associated with the reef structure or matrix and are prone to predation pressure following coral loss and the longer-term loss of the physical structure of the reef matrix (Munday & Jones 1998), consistently displaying greater population declines than larger-bodied counterparts (Graham *et al.* 2008). All four of these variables have consistently been shown to relate to population declines following coral habitat disturbances in a range of empirical studies (reviewed by Wilson *et al.* 2006; Pratchett *et al.* 2008). These four variables were quantified for 134 species, including their juvenile and adult life stages, using extensive searches of the literature, FishBase and expert knowledge. It is important to note that other impacts on coral reef habitat, such as crown-of-thorns starfish outbreaks, disease and storm damage, typically cause coral mortality in a similar way to coral bleaching and impacts on fish are likely to be closely aligned with the climate vulnerability axis described here (Wilson *et al.* 2006). Other life-history traits, such as age at maturity, were not included in this composite indicator because climate-driven coral bleaching events are pulse disturbances with declines associated with specialization and body size (Pratchett *et al.* 2008). Alternative life-history traits are more appropriate for assessing vulnerability to press disturbances, such as fishing (Cheung *et al.* 2005).

The extinction risk index was based on three established indicators of extinction risk; geographical range size, occupancy and numerical rarity (Rabinowitz 1981; Gaston 1991) and one specific to coral reef fishes, depth range. Each species’ geographical range size was calculated as polygons of coastal waters surrounding reefs where the species are present (Allen 2008). Areas of open ocean between reefs that are geographically close are included in polygons, but large expanses of open ocean, for example between Sri Lanka and Indonesia, are not included (Allen 2008). Occupancy was based on the presence of a species in a geographical location from 66 surveys in the mid-1990s across the Indian Ocean (Graham *et al.* 2008). Where a species was present at one or more sites, the area of that geographical location was included in the calculation of occupancy. This method was chosen over simply totalling the number of sites at which a species was present, due to occupancy-abundance relationships, which can confound the use of both occupancy and numerical rarity (He & Gaston 2008). The occupancy method used here had a correlation coefficient with numerical rarity of 0.27, suggesting little collinearity between the two indicators. These mid-1990s Indian Ocean data are from before the region was impacted by the 1998-bleaching event, which non-randomly altered the occupancy and abundance of reef fish species (Graham *et al.* 2008). Numerical rarity was calculated using area-standardized abundance data from the same mid-1990s dataset, for geographical locations where the species occurred. Depth range was included in our measure of extinction risk for reef fishes because most threats, including coral bleaching and fisheries exploitation, occur, or are most severe, at shallow depths on coral reefs (Sheppard & Obura 2005; Tyler *et al.* 2009). Therefore, part of the population of fish species with extended depth ranges will likely not be impacted by a given disturbance. Depth range data were collated using the best available information from FishBase and the literature, although the depth distribution of some species can be deeper than expected due to sampling constraints.

The four variables making up both the climate change vulnerability and extinction risk composite indices were combined using the expert ranked weighting scheme, or Analytic Hierarchy Process (AHP; Saaty 1980). Each variable was scaled from 0 to 1, prior to weighting and combining. Eight scientific experts (five of the authors and three independent experts, all of whom have broad experience from a range of geographical locations) individually made pair-wise comparisons of the importance of each of the two sets (climate vulnerability and extinction risk) of four variables. In each pair-wise assessment, the expert provided a score indicating whether the variables had equal importance (score 1), one was slightly more important that the other (score 2), or one was much more important that the other (score 3). A weighting value was calculated for each variable from the resultant matrix using AHP and applied to the scaled data for each variable. Bray–Curtis similarity indices between the different researchers’ weightings ranged from 59 to 94 for climate change vulnerability and 59–96 for extinction risk, indicating general consensus regarding the weighting. The resultant weighting for the climate vulnerability axis was 0.33 for settlement specialization, 0.28 for dietary specialization, 0.24 for habitat specialization and 0.15 for body size. The weighting for the extinction risk axis was 0.29 for geographical range size, 0.25 for occupancy, 0.25 for depth range and 0.20 for numerical rarity. These weightings were used to calculate the final indices for each species based on the weighted sum of the four variables. An independent assessment of the four climate vulnerability variables using the Seychelles dataset described below, confirmed the weighting assigned by the experts (see Table S1), whereby settlement specialization had the strongest relationship to the data, body size the weakest and diet and habitat specialization fell in the middle. These weights controlled the contribution of each variable to species level vulnerability and down weighted the higher proportion of species with small body size that occurs at the community scale (Munday & Jones 1998).

The four focal families of the study were the Acanthuridae (surgeonfishes), Scaridae (parrotfishes), Chaetodontidae (butterflyfishes) and Labridae (wrasses). These four families were chosen, as they are common and specious groups that represent a range of body sizes, functional groups and fisheries importance on coral reefs. Furthermore, all species present were comprehensively surveyed within these four families at each location in the mid-1990s Indian Ocean survey and thus, the issue of truncated species lists in surveys was not of concern. A total of 134 species were included within these four families, which represented eight functional groups. Species were assigned to the functional groups defined in Wilson *et al.* (2008) using the literature and FishBase. This categorization of functional groups includes information on trophic guilds, dietary specialization and life-history characteristics.

Species positions on the bivariate plot of the climate change and extinction risk indices predict the likelihood of population decline and whether such a decline could be catastrophic for species global persistence (Fig. 1a). In such a framework, a species falling in the upper right quadrant is predicted to be susceptible to population declines following coral reef climatic disturbance events. Such population declines are likely to be catastrophic due to a small geographical range size, shallow depth range, low natural abundance and limited occupancy. These species are predicted to be at greatest risk of global extinction from climate change impacts to coral reefs. Species falling in the upper left quadrant of the figure are expected to be prone to local or regional extinctions following large-scale disturbances. Species falling in the lower quadrants are unlikely to display large population declines in response to climate disturbances on reefs. However, species in the lower right quadrant may be extinction prone due to other disturbances or stochastic population variation.

To assess the occurrence of population declines due to multiple jeopardy in response to climate disturbances, we produced a Venn diagram of the four indicators used in the composite climate vulnerability index. Using the scaled data for each indicator, we assigned a species as having a high vulnerability to that indicator if it had a score > 0.7, which is conservative given that all species declined in the Seychelles assessment with a combined indicator score > 0.6 (see Results). The Venn diagram allows all possible single, double, triple or quadruple jeopardy combinations of the indicators to be clearly represented.

An assessment of the predictive ability of the climate vulnerability index was conducted using data from the inner Seychelles islands. These were the best data available to assess the index because they covered a large area, had high levels of replication and the location suffered the greatest recorded coral bleaching disturbance to date (> 90% coral lost in 1998). Highly variable impacts of bleaching mortality over larger geographical areas precluded a broader assessment (e.g. Graham *et al.* 2008). Twenty-one sites, covering over 50 000 m^2^ of coral reef habitat, were surveyed at the same time of year in 1994 and 2005. At each site, 16 replicate 7 m radius point counts were completed using underwater visual census along the base of the reef slope. The numerical abundance of diurnally active, non-cryptic, juvenile and adult reef fishes > 8 cm (TL) was estimated within each count area. For full survey method details, see Graham *et al.* (2007). The per cent change in abundance was calculated for each species between 1994 and 2005. As per cent differences can have a strong right-tailed distribution, i.e. a maximum potential decline of 100%, but potentially limitless increases, we transformed data following [*Y* = log_e_(1 + [Δ/101])]. The transformation approximately normalizes the error distribution and stabilizes its variance with the data balanced around zero and a common maximum decline and increase of −4.6 and +4.6 imposed. A Bayesian normal linear model was fit to the climate vulnerability index and species population decline data, with 95% posterior credible and predictive intervals used to assess the strength of the linear relationship (Table 1a). Uninformative model priors were N(0, 0.001) and Γ(0.001, 0.001) for coefficients and variances respectively. Model fit was assessed using Bayesian goodness-of-fit (GOF) values, whereby deviations of observed data from model simulated values provide substantial evidence of poor model fit at large (> 0.975) or small (< 0.025) GOF values (Gelman *et al.* 1996).

**Table 1:** Posterior parameter estimates for (a) the relationship between climate change vulnerability and pre-/post-bleaching change in fish abundance among sites in the Seychelles and (b) the relationship between fishing vulnerability and climate change vulnerability

Parameter	Mean	Median	95% CI
(a)			
β_0_	0.79	0.81	0.13, 1.43
β_1_	−3.24	−3.25	−4.71, −1.78
τ	0.91	0.89	0.58, 1.30
(b)			
β_0_	5.04	5.02	3.52, 6.57
β_1_	−7.65	−7.64	−14.52, −1.00
β_2_	1.12	1.16	−5.09, 7.64
τ	0.29	0.32	0.23, 0.36

Coefficients in (a) are for a normal linear-model fit and in (b) for a second-order polynomial fit using a log-Normal distribution; τ is the posterior precision; CIs are Bayesian posterior 95% credible intervals; β values not overlapping zero provide strong evidence of a positive or negative relationship.

To assess the combined effects of climate change and fisheries, values for species vulnerability to fishing were extracted from Cheung *et al.* (2005). This fisheries vulnerability indicator uses a fuzzy logic expert system to take account of eight life-history characteristics that make species vulnerable to fisheries exploitation. The indicator has good predictive power (Cheung *et al.* 2005) and has been widely recognized as a comprehensive and suitable indicator of the vulnerability of fish species to fishing (Reynolds *et al.* 2005). The relationship between fishing vulnerability and climate vulnerability was assessed using a Bayesian log-Normal model to account for the skewed variation in the data towards the fishing vulnerability axis and the positive fishing vulnerability response (Table 1b), with priors and model fit defined as for the linear model above. Deviance information criteria (DIC), based on a log-Normal distribution, favoured a second-order polynomial model (DIC = 1183.26) over a standard linear model (DIC = 1207.04) where DIC values > 2 provide substantial support for the lowest-DIC model. Extinction risk was represented by bubbles, with increasing size related to increasing risk. This methodology was used as the clearest way to visually assess the spread of extinction prone species across the range of fisheries exploitation and climate change driven habitat degradation values. As body size was included in both the vulnerability to climate disturbances and fishing, we repeated the analysis when body size was removed from the climate vulnerability axis.

## Results

Reef fish functional groups were clearly segregated along the climate vulnerability axis (Fig. 1b). The functional group predicted to be most vulnerable was the obligate corallivores, followed by facultative corallivores and micro-invertivores. The groups predicted to be least vulnerable were the macro-invertivores and the roving, scraping and excavating herbivores.

All functional groups had a broad spread along the extinction risk axis, with species in each group having low and high risks of extinction (Fig. 1b). The species we predict to be most vulnerable to population declines following climatic reef disturbances is the tubelip wrasse, *Labrichthys unilineatus* (Table S2). This small-bodied, highly specialized species settles, inhabits and feeds only on corals. However, *L. unilineatus* has a medium global extinction risk score; although it has a fairly shallow depth range and occurs in relatively low abundance, it is commonly found at most reef locations (i.e. high occupancy) and has a broad geographical range. In such a case, we predict local rather than global extinction is more likely. *Chaetodon triangulum*, which fell closest to the top right corner of Fig. 1b, has a relatively high climate vulnerability and extinction risk score (Table S2). This species depends on living corals, is commonly found in low numbers, is not present in all locations within its range, is restricted to the Indian Ocean and has a depth range limited to 25 metres.

Fifty-six of the 134 species investigated had a high vulnerability to climate change attributed to at least one of the indicators used (Fig. S2). Seven of the eight functional groups included were represented by these high vulnerability species, with only species from the scraper/excavator group showing moderate vulnerability to either indicator. The majority of these were small-bodied species. All species with highly specialized diet and habitat requirements have either settlement or body size attributes that contribute to their high level of climate change susceptibility.

**Figure 2:**
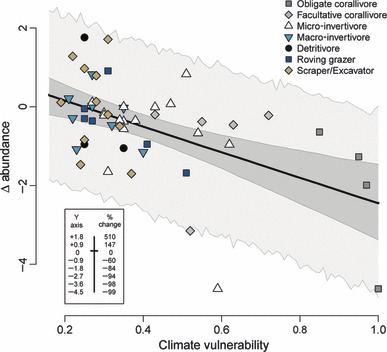
Assessing the climate vulnerability index through a major disturbance event. Predicted climate change vulnerability scores and fish species population change in the inner Seychelles through the 1998 bleaching event. Trend line represents a Bayesian normal linear-model fit, with the posterior 95% credible and predictive intervals represented as dark and light grey shading respectively.

Plotting the predicted climate change vulnerability index with observed population changes through the 1998 coral bleaching event in the inner Seychelles islands produced a strongly negative relationship that was well-fit by a normal linear model (GOF = 0.263), whereby species with a higher vulnerability score displayed the greatest declines in abundance (Table 1a, Fig. 2). Lending further support to our predictive framework, any species with a vulnerability score > 0.6 experienced a decline in abundance and species of obligate corallivores, facultative corallivores and micro-invertebrate feeders (the groups predicted as most vulnerable in Fig. 1b) all declined.

**Figure 3:**
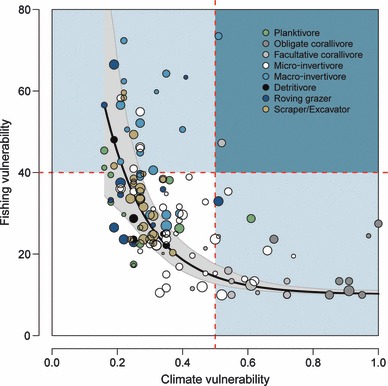
Relationship between vulnerability of coral reef fish species to climate change disturbance (i.e. bottom-up habitat degradation) and fisheries (i.e. top-down exploitation). The nonlinear negative relationship represents stress-induced community sensitivity, whereby the species pool is greatly reduced if stressors co-occur. The size of the bubbles is proportional to extinction risk. Trend line represents a Bayesian log-Normal second-order polynomial fit with the 95% credible interval represented as grey shading. Blue shading represents hypothetical stress levels.

There is a clearly negative and steeply convex relationship in species vulnerabilities to the multiple threats of climate change driven habitat loss and fisheries exploitation (GOF = 0.890), whereby those species most vulnerable to climatic disturbances are least vulnerable to fishing and vice versa (Table 1b, Fig. 3). Importantly, when body size was removed from the climate vulnerability axis, a second-order polynomial model was still the best fit to the data and the confidence intervals overlap considerably with the plot of the data including body size (Fig. S1). Therefore, the trend is not solely driven by body size although body size is clearly an ecologically important variable to the individual vulnerability of both stressors. The functional groups most affected along the climate change axis are obligate corallivores, facultative corallivores and micro-invertebrate feeders (Fig. 3). The functional groups most vulnerable to fishing are macro-invertebrate feeders, scrapers/excavators, roving grazers and some micro-invertebrate feeders. Encouragingly, most functional groups are represented by some species in the bottom left corner of the plot, where vulnerability to both stressors is likely to be low. The only group not represented in the bottom left corner of the plot is the obligate corallivores. Extinction risk is spread evenly along both axes (bubble size in Fig. 3).

## Discussion

This study brings together information from life-history traits, expert opinion and distribution patterns to create a unique assessment of extinction vulnerability. The approach enables a predictive assessment of the taxa or functional groups most at risk of local or global extinction. We applied the framework to the threat climate change poses to coral reef fish assemblages, determining that our climate change vulnerability axis had good predictive power when assessed against data before and after a severe coral bleaching event in the inner Seychelles. Furthermore, the predicted vulnerable functional groups are also those that show greatest declines in meta-analyses of studies assessing the impacts of coral mortality on fishes (Wilson *et al.* 2006; Pratchett *et al.* 2008). Importantly, the framework has great generic value and can be readily applied to organisms other than coral reef fishes and to stressors other than climate change.

Among reef fish we found some functional groups were more vulnerable to population declines through climatic disturbance, however no single functional group was more prone to global extinction. We identified obligate corallivores as the most vulnerable functional group to climate disturbances. This result was expected given the variables used in our predictive framework and based on a wide literature (Pratchett *et al.* 2008). However, many species of obligate corallivore had broad geographical distributions and high occupancies. This finding suggests that although local abundance of obligate corallivores may decline following climate disturbances, stocks in adjacent reefs may foster recovery. Critically however, obligate corallivorous species were highly clustered in their vulnerability to climate disturbances, reflecting a low diversity of responses locally to disturbance as a functional group (Vinebrooke *et al.* 2004). Such low response diversity suggests that obligate corallivores may lose all representative species locally from a heavily disturbed location. The effects of these declines on the functioning of reef ecosystems are thought to be small as obligate corallivores are not highly abundant on coral reefs and the energy pathway from corals to fish is apparently weak (Cole *et al.* 2008). Facultative corallivores and micro-invertebrate feeders were the next groups most vulnerable to climate disturbance. These functional groups are predominately composed of small-bodied fish that are vulnerable to longer-term declines in the topographical complexity of the reef (Graham *et al.* 2006). While these groups are important for transferring energy through the foodweb, they are not thought to be critical functional groups that maintain ecosystem functions on coral reefs (Bellwood *et al.* 2004).

Conversely, roving grazers and scraper/excavators, more vulnerable to the effects of fishing than habitat loss, have been identified as key functional groups on coral reefs (Bellwood *et al.* 2004), controlling proliferation of benthic algae that affects coral recruitment and recovery processes after disturbance (Hughes *et al.* 2007; Mumby *et al.* 2007). The low vulnerability of roving grazers and scraper/excavators to climate-induced reef disturbance suggests that key functions can be maintained after such events. After very severe disturbances where the reef structure collapses, small to medium size classes of these groups can decline in abundance due to exposure to predators (Graham *et al.* 2007). There are, however, considerable time lags involved, indicating that key functions can continue for decades (Graham *et al.* 2007) and promote recovery processes. Macro-invertivores also had a low vulnerability to climate disturbances, which is important because these species are important for controlling large invertebrates including coral-eating starfish and bioeroding sea urchins (McClanahan 2000; Dulvy *et al.* 2004).

The species that we determined was most vulnerable to population declines following climatic reef disturbance was the tubelip wrasse, *L. unilineatus*. Indeed, the local extinction of *L. unilineatus*, along with three other species, was reported from the inner Seychelles following the 1998 coral bleaching event (Graham *et al.* 2006) and any recovery will depend on a combination of habitat regeneration and connectivity to unaffected populations. However, *C. triangulum* had the highest relative vulnerability to global extinction, due to low occupancy, low numerical abundance and restricted geographical and depth ranges. The majority of the species with high vulnerability to extinction were small-bodied because diversity peaks in small size classes in coral reef fishes (Munday & Jones 1998). Loss of reef structure following disturbances reduces predator-free space for small fishes (Munday & Jones 1998), making them highly vulnerable to population declines (Graham *et al.* 2006). Conversely, the contribution of diet, habitat and settlement specialists to a coral reef fish community can be relatively small (Pratchett *et al.* 2008). Interestingly, 37% of the 56 high vulnerability species had two or more attributes that made them susceptible to the effects of climate-induced coral bleaching, demonstrating that many species face multiple risks of population decline. It should be noted that because scientists still have a lot to learn regarding species life histories and behaviours, there will be surprises regarding which species survive in a changing climate.

Along with climate change driven habitat loss (Hoegh-Guldberg *et al.* 2007), the other greatest threat to coral reef fishes is fisheries exploitation (Halpern *et al.* 2008). We found a strongly convex relationship among the species affected by these two stressors, whereby species vulnerable to one threat are unlikely to be affected by the other. This finding reduces the possibilities of strong synergistic effects of fishing and climate change at a species level. However, the convex relationship between fishing and climate susceptibility indicates extreme sensitivity at a community level (Vinebrooke *et al.* 2004). Biodiversity of all functional groups is likely to be severely reduced if both stressors are present, because species that survive one of the stressors will, on average, be vulnerable to the other. At a community level, such relationships represent additive, rather than synergistic, forces on composition and associated function (Vinebrooke *et al.* 2004; Darling & Côté 2008). An increased severity of the disturbances (increasing the shaded areas in Fig. 3) will not result in many individual species being affected by both stressors, but larger portions of the community will be affected by one or the other stress. This phenomenon is a sobering finding, because such multi-stress community reductions are likely to be common as there are few coral reefs around the world that are not affected by fishing pressure (Halpern *et al.* 2008) and climate change is an increasingly dominant driver of coral reef decline (Hoegh-Guldberg *et al.* 2007).

The increased presence of multiple stressors on coral reefs raises the potential for feedbacks between impacts such as climate and fishing. For example, the longer-term effects of extreme habitat degradation following coral bleaching can lead to declines in smaller cohorts of larger fishery target species, or a reduction in their prey (Graham *et al.* 2007). Fishing may lead to degradation of benthic habitat through direct damage by destructive gears (McClanahan *et al.* 2008) or through trophic cascades (Mumby *et al.* 2007), both of which are likely to effect habitat dependent reef fish. Furthermore, predatory release of large invertebrates, such as coral feeding crown-of-thorns starfish (Dulvy *et al.* 2004) and bioeroding sea urchins (McClanahan 2000), can have profound local impacts on benthic communities. We were unable to incorporate such interactions into our framework, but they clearly need to be considered where fishing or benthic disturbances are severe.

Studies in other ecosystems have also identified differential sensitivity to multiple stressors (Crain *et al.* 2008; Darling & Côté 2008). For example, an assessment of extinction risk in birds demonstrated that small, specialized bird species were most vulnerable to habitat loss, whereas large-bodied species with long generation times were most vulnerable to predation and persecution (Owens & Bennett 2000). Response of primate communities to multiple extinction threats is also non-random and clearly segregated; large primates are vulnerable to hunting, specialized species are vulnerable to forestry and species that occupy niches low in the canopy are most vulnerable to agriculture (Isaac & Cowlishaw 2004). The framework presented in our study builds on these contributions by separating predictors of population decline from extinction risk and assessing the impacts of multiple stressors on distinct functional groups within the community.

In terms of ecosystem function, we found that key functional groups of fish were aligned with the fishing vulnerability axis. Fishing on coral reefs is typically multi-species and size based, which, along with key life-history traits, makes these functional groups vulnerable to fishing (Cheung *et al.* 2005). For example, the roving grazers and scraper/excavators that control algal growth (Bellwood *et al.* 2004) are more susceptible to the effects of fishing than climate. Similarly, some macro-invertivore feeding species, targeted by fishers, are important in controlling outbreaks of mobile invertebrates on reefs and are often numerically abundant in the absence of heavy exploitation (McClanahan *et al.* 2007). Therefore, fishing vulnerability is of particular concern, because common species in key groups often exert the greatest role in ecosystem stability and function (Smith & Knapp 2003; Gaston & Fuller 2007). Piscivores were the main functional group missing from our analysis because the main family comprising piscivorous species (Serranidae), and those containing macro-carnivores (e.g. Lethrinidae and Lutjanidae), were not consistently sampled across the Indian Ocean in the mid-1990s dataset. However, it is highly likely that these groups would align with the fishing axis and not the climate vulnerability axis. This alignment is because piscivores and macro-carnivores are among the most vulnerable groups to fishing (Cheung *et al.* 2005), but are not specialized on live coral for diet or habitat (Pratchett *et al.* 2008) and larvae tend to settle into non-coral habitats, such as rubble areas (Light & Jones 1997).

Alignment of functionally important fish with the fishing vulnerability axis has positive ramifications in terms of policy. Climate change is a global-scale issue, with committed increases in mean global temperature owing to existing and ongoing emissions of greenhouse gases. Accordingly, the effects of any policies to mitigate greenhouse gas emissions will be slow and impacts on coral reefs will continue even if emissions are stabilized (Hoegh-Guldberg *et al.* 2007). Conversely, fishing is predominantly governed by local-scale demand and policies so responses to management are more rapid. Increases in reef fish biomass, including the biomass of important functional groups described here, can be achieved using a range of measures that include catch and effort controls (Mapstone *et al.* 2008), gear restrictions (McClanahan *et al.* 2008), no-take marine protected areas (Stockwell *et al.* 2009) and economic development of coastal communities (Cinner *et al.* 2009). It is encouraging that increases in the abundance of some of these functional groups can support recovery between climate-driven coral bleaching events (Hughes *et al.* 2007; Mumby *et al.* 2007) and thus buffer the negative effects of climate warming. Taking the fishery management action needed to achieve the recovery of reef fish populations across the seascape will be challenging, but it will provide substantial ecosystem and resource benefits, buying much needed time to address the more complex global problem of reducing atmospheric CO_2_ levels.

## SUPPORTING INFORMATION

**Figure S1** Vulnerability of coral reef fish species to climate change disturbance and fisheries with body size removed from climate vulnerability axis.

**Figure S2** Multiple jeopardy of extinction.

**Table S1** Independent test of climate vulnerability variables using Seychelles dataset.

**Table S2** Twenty most vulnerable coral reef fish species surveyed to climate change disturbances.

As a service to our authors and readers, this journal provides supporting information supplied by the authors. Such materials are peer-reviewed and may be re-organized for online delivery, but are not copy-edited or typeset. Technical support issues arising from supporting information (other than missing files) should be addressed to the authors.
