# Supplementary material for: Extinction vulnerability of coral reef fishes
Source: Ecol Lett. 2011 Apr;14(4):341–8. doi: 10.1111/j.1461-0248.2011.01592.x (PMC3627313; doi:10.1111/j.1461-0248.2011.01592.x)
Supplement: Supplementary file 1 [file ele0014-0341-SD1.doc]

**SUPPORTING INFORMATION**

**Extinction vulnerability of coral reef fishes**

Nicholas A. J. Graham, Pascale Chabanet, Richard D. Evans, Simon Jennings, Yves Letourneur, M. Aaron MacNeil, Tim R. McClanahan, Marcus C. Öhman, Nicholas V. C. Polunin and Shaun K. Wilson

The following Supporting Information is available for this article:

**Table S1** Independent test of climate vulnerability variables using Seychelles dataset.

**Table S2** Twenty most vulnerable coral reef fish species surveyed to climate change disturbances.

**Figure S1** Vulnerability of coral reef fish species to climate change disturbance and fisheries, with body size removed from climate vulnerability axis.

**Figure S2** Multiple jeopardy of extinction.

Additional Supporting Information may be found in the online version of this article.

Please note: Blackwell Publishing is not responsible for the content or functionality of any supporting information supplied by the authors. Any queries (other than missing material) should be directed to the corresponding author for the article.

**Table S1** Independent test of climate vulnerability variables using Seychelles dataset. Test statistics represent individual linear regression analyses.

| **Variable** | **Expert weight** | **Test on Seychelles data** | | |
| --- | --- | --- | --- | --- |
|  |  | *R^2^* | *F* | *P* |
| Body size | 0.15 | 0.11 | 6.49 | 0.014 |
| Habitat | 0.24 | 0.17 | 10.40 | 0.002 |
| Diet | 0.28 | 0.14 | 8.30 | 0.006 |
| Settlement | 0.33 | 0.25 | 16.81 | <0.000 |

**Table S2** Twenty most vulnerable coral reef fish species surveyed to climate change disturbances. Species listed in decreasing order of vulnerability. Maximum scores for both climate change vulnerability and extinction risk indicators =1.

| **Family** | **Species** | **Functional group** | **Climate change vulnerability** | **Extinction risk** |
| --- | --- | --- | --- | --- |
| Labridae | *Labrichthys unilineatus* | Obligate corallivore | 1.00 | 0.64 |
| Chaetodontidae | *Chaetodon trifascialis* | Obligate corallivore | 0.97 | 0.38 |
| Chaetodontidae | *Chaetodon trifasciatus* | Obligate corallivore | 0.95 | 0.60 |
| Chaetodontidae | *Chaetodon meyeri* | Obligate corallivore | 0.91 | 0.63 |
| Chaetodontidae | *Chaetodon triangulum* | Obligate corallivore | 0.91 | 0.86 |
| Chaetodontidae | *Chaetodon bennetti* | Obligate corallivore | 0.88 | 0.63 |
| Chaetodontidae | *Chaetodon zanzibariensis* | Obligate corallivore | 0.85 | 0.71 |
| Labridae | *Pseudocheilinus hexataenia* | Micro-invertivore | 0.74 | 0.35 |
| Chaetodontidae | *Chaetodon citrinellus* | Facultative corallivore | 0.72 | 0.63 |
| Chaetodontidae | *Chaetodon guttatissimus* | Facultative corallivore | 0.72 | 0.60 |
| Chaetodontidae | *Chaetodon unimaculatus* | Facultative corallivore | 0.72 | 0.51 |
| Labridae | *Labropsis xanthonota* | Obligate corallivore | 0.68 | 0.70 |
| Chaetodontidae | *Chaetodon falcula* | Micro-invertivore | 0.66 | 0.76 |
| Chaetodontidae | *Chaetodon kleinii* | Facultative corallivore | 0.63 | 0.35 |
| Chaetodontidae | *Chaetodon xanthocephalus* | Micro-invertivore | 0.62 | 0.78 |
| Acanthuridae | *Paracanthurus hepatus* | Planktivore | 0.61 | 0.70 |
| Chaetodontidae | *Chaetodon collare* | Facultative corallivore | 0.61 | 0.94 |
| Labridae | *Thalassoma hardwicke* | Micro-invertivore | 0.59 | 0.52 |
| Chaetodontidae | *Chaetodon lunula* | Facultative corallivore | 0.55 | 0.46 |
| Chaetodontidae | *Chaetodon madagaskariensis* | Facultative corallivore | 0.55 | 0.62 |


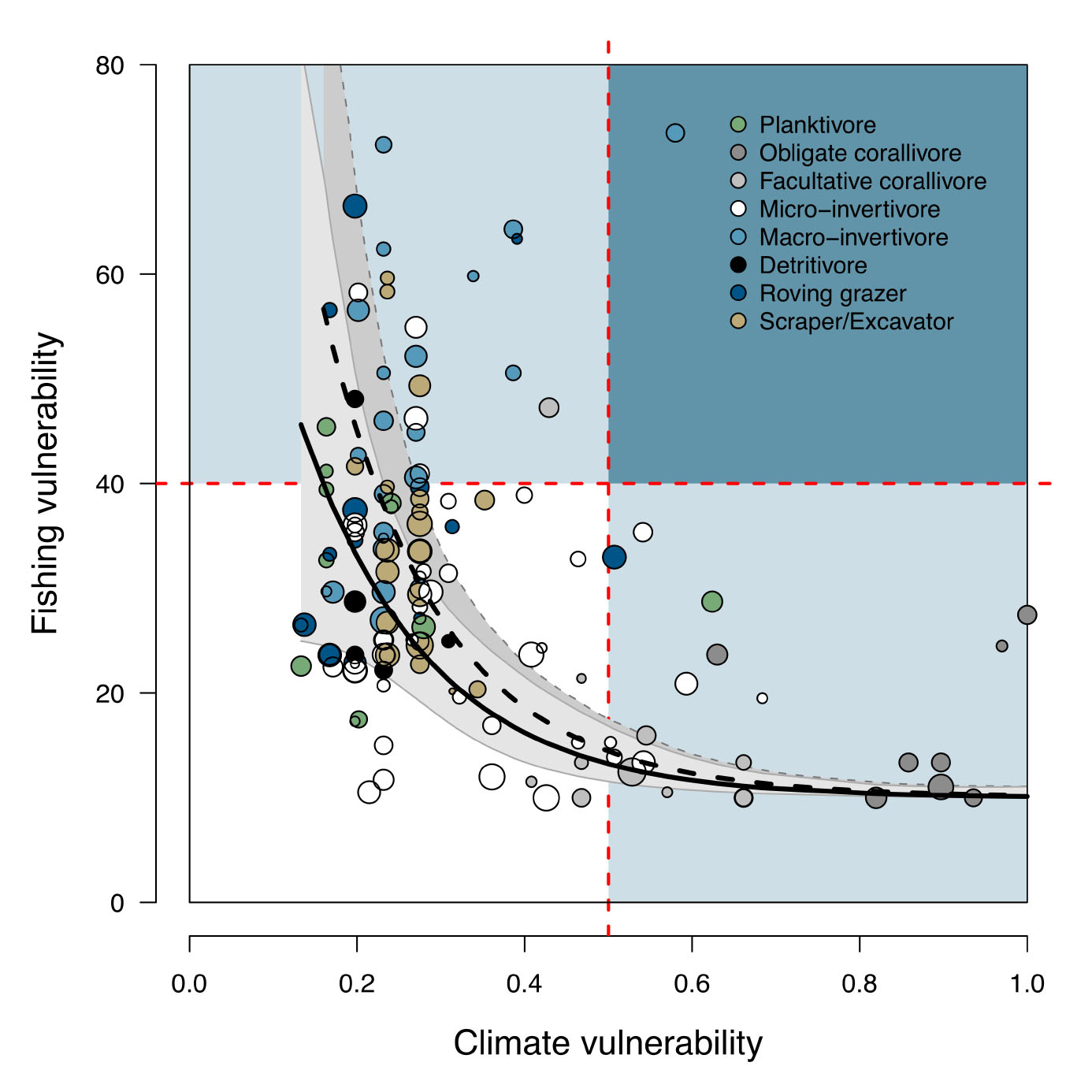


**Figure S1** Vulnerability of coral reef fish species to climate change disturbance and fisheries, with body size removed from climate vulnerability axis. Trend lines represents Bayesian log-Normal 2^nd^-order polynomial fits, with the 95% credible intervals represented as grey shading. The dashed trend line is for the data including body size in the climate vulnerability axis (i.e. the data presented in Fig. 3) and the solid trend line is for the data presented here with body size removed from the climate vulnerability axis. The size of the bubbles is proportional to extinction risk. Blue shading represents hypothetical stress levels.


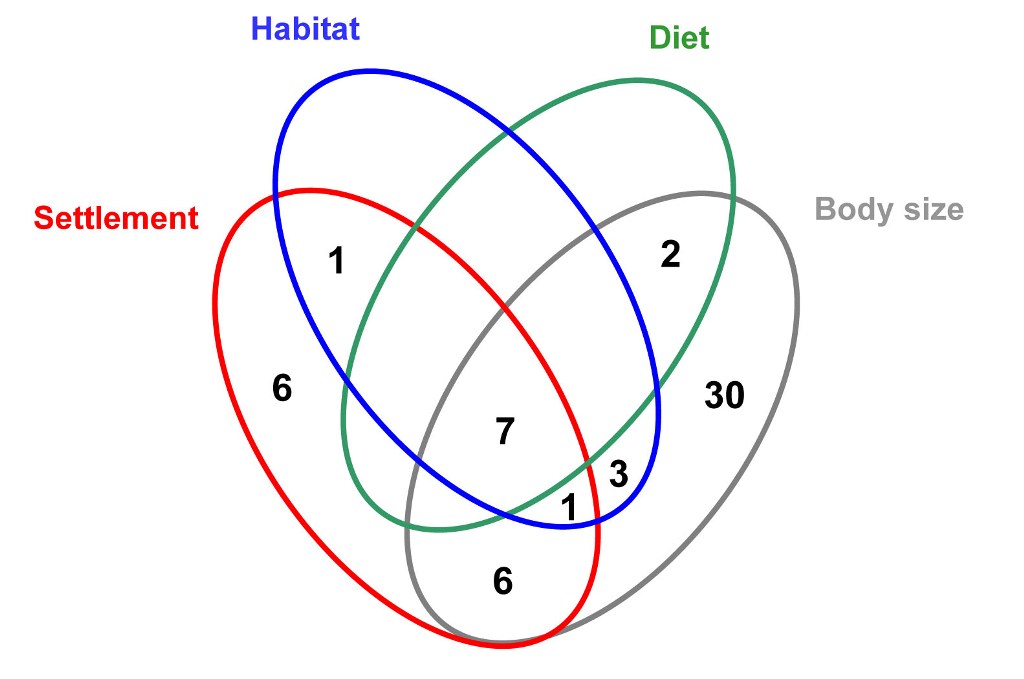


**Figure S2** Multiple jeopardy of extinction. Venn diagram of the four indicators used to predict fish species vulnerability to coral bleaching events. Of the 134 species included in the analysis, 56 had a high vulnerability to at least one of the indicators, and 7 had attributes lending them to quadruple jeopardy of population decline.
